# Lignin-Based Nanostructured Sensor for Selective Detection of Volatile Amines at Trace Levels

**DOI:** 10.3390/s25113536

**Published:** 2025-06-04

**Authors:** Paolo Papa, Giuseppina Luciani, Rossella Grappa, Virginia Venezia, Ettore Guerriero, Simone Serrecchia, Fabrizio De Cesare, Emiliano Zampetti, Anna Rita Taddei, Antonella Macagnano

**Affiliations:** 1 Institute of Atmospheric Pollution Research (IIA) of National Research Council (CNR), Montelibretti, 00010 Rome, Italy; ettore.guerriero@cnr.it (E.G.); simoneserrecchia@cnr.it (S.S.); decesare@unitus.it (F.D.C.); emiliano.zampetti@cnr.it (E.Z.); 2 Department of Chemical, Materials and Production Engineering (DICMaPI), University of Naples Federico II, 80125 Naples, Italy; luciani@unina.it (G.L.); rossella.grappa@unina.it (R.G.); virginia.venezia@unina.it (V.V.); 3 Department for Innovation in Biological, Agro-Food and Forest Systems (DIBAF), University of Tuscia, 01100 Viterbo, Italy; 4 High Equipment Centre, Electron Microscopy Section, University of Tuscia, Building D, 01100 Viterbo, Italy; artaddei@unitus.it

**Keywords:** lignin nanoparticles, polylactic acid, amine sensing, dimethylamine, electrospinning, VOCs sensor, selective sensor

## Abstract

A nanostructured sensing platform was developed by integrating gold-decorated lignin nanoparticles (AuLNPs) into electrospun polylactic acid (PLA) fibre mats. The composite material combines the high surface-to-volume ratio of PLA nanofibres with the chemical functionality of lignin—a polyphenolic biopolymer rich in hydroxyl and aromatic groups—enabling selective interactions with volatile amines through hydrogen bonding and Van der Waals forces. The embedded gold nanoparticles (AuNPs) further enhance the sensor’s electrical conductivity and provide catalytic sites for improved analyte interaction. The sensor exhibited selective adsorption of amine vapours, showing particularly strong affinity for dimethylamine (DMA), with a limit of detection (LOD) of approximately 440 ppb. Relative humidity (RH) was found to significantly influence sensor performance by facilitating amine protonation, thus promoting interaction with the sensing surface. The developed sensor demonstrated excellent selectivity, sensitivity and reproducibility, highlighting its potential for real-time detection of amines in environmental monitoring, industrial safety and healthcare diagnostics.

## 1. Introduction

The development of advanced gas sensors based on nanostructured materials has emerged as a critical area of research due to the increasing demand for real-time, low-cost and highly sensitive devices for environmental monitoring, industrial safety, and healthcare diagnostics. Nanomaterials, owing to their high surface area, tunable physicochemical properties and enhanced reactivity, offer significant advantages in improving sensor sensitivity, selectivity and response time [1].

Volatile amines, including primary, secondary and tertiary species, such as dimethylamine (DMA), trimethylamine (TMA) and ammonia, represent an important subclass of volatile organic compounds (VOCs) with significant implications across multiple fields. In the food industry, these compounds are recognized as early spoilage indicators, especially in protein-rich products, such as fish, meat and dairy, where microbial degradation leads to biogenic amine accumulation [2,3]. Their quantification is crucial for assessing freshness, ensuring food safety and complying with regulatory thresholds. In agriculture and livestock management, amines and ammonia emissions result from fertilizer application and manure decomposition, contributing not only to environmental pollution (e.g., eutrophication, acidification) but also to occupational health risks [4]. In medical diagnostics and forensic science, certain amines serve as biomarkers for metabolic disorders, bacterial infections, or post-mortem degradation, thus supporting disease diagnosis or time-of-death estimations. Given their physiological and environmental significance, the real-time, selective detection of volatile amines at trace levels has become a priority for developing next-generation chemical sensors. Sensors capable of operating under ambient conditions with high sensitivity and rapid response are increasingly sought for integration into food packaging, wearable healthcare devices and environmental monitoring systems [5,6,7].

In fact, the accumulation of amines in air poses significant health risks, such as respiratory irritation and neurological effects [8]. Moreover, amines and ammonia compounds are valuable indicators in various environmental and biological processes, making their detection crucial for applications such as air quality monitoring, food safety, and industrial hygiene. Although volatile amines are increasingly recognized as critical markers in environmental and food safety monitoring, sensor development for their selective and low-level detection still lags behind that of other VOCs, such as aldehydes and aromatic hydrocarbons, particularly in terms of commercially available solutions.

Conventional methods of amine detection, such as gas chromatography and mass spectrometry, while highly sensitive, are costly and not suitable for real-time, on-site applications. Gas sensors based on nanomaterials have emerged as a promising alternative, offering real-time, cost-effective, and portable solutions for detecting amines and other VOCs [9]. However, the challenge of designing sensors with high sensitivity and selectivity for specific VOCs, particularly amines, is still a significant research gap. Traditionally, a wide range of synthetic polymers have been employed in gas sensor design, either as standalone sensing layers or as matrices for the incorporation of functional inorganic and organic additives. These materials can be engineered into various hybrid or composite nanostructures capable of detecting gaseous analytes across a broad concentration range [10,11,12,13]. However, increasing concerns over the environmental footprint of synthetic polymers—particularly their persistence and low degradability—have driven the research community to seek sustainable alternatives [14,15]. In response to these challenges, the development of bio-based sensing materials derived from renewable or waste resources is gaining momentum [16,17]. Among them, lignin—a complex aromatic biopolymer and the second most abundant natural polymer on Earth—stands out due to its unique combination of biodegradability, chemical versatility, antioxidant properties and availability as a byproduct of the pulp and paper industry [18]. Recent studies have highlighted the potential of lignin as a sensing material owing to its intrinsic reactivity with various gaseous species and its capacity to be functionalized or hybridized with other components to form advanced composites [19,20,21]. Furthermore, lignin nanoparticles can address poor reproducibility and heterogeneity issues related to lignin source and extraction methods [22].

In this work, we report for the first time the design of a fully biodegradable and sustainable gas sensor composed of electrospun polylactic acid (PLA) nanofibres functionalized with gold-decorated lignin nanoparticles (PLA-AuLNPs), enabling the selective detection of volatile amines under ambient conditions. PLA, a well-known biodegradable and biocompatible polymer derived from renewable resources, serves as an ideal scaffold due to its ease of electrospinning, mechanical robustness, and environmental friendliness [23]. Nevertheless, its inherent electrical insulation limits its standalone application in chemosensing [24]. To overcome this limitation, we introduce a green synthesis route in which lignin nanoparticles act simultaneously as reducing and stabilizing agents for the in situ formation of gold nanoclusters, eliminating the need for external chemical reductants. By exploiting the reducing properties of lignin, nanostructured metallic gold was obtained directly from an aqueous solution of tetrachloroauric acid trihydrate (HauCl_4_∙3H_2_O), leading to the in situ formation of Au^0^ on the surface of lignin nanoparticles. This process allowed the lignin nanoparticles to be effectively decorated with gold, forming hybrid LNPs-AuNPs without the need for external chemical reducing agents. This eco-friendly strategy not only minimizes synthetic complexity and toxicity but also ensures a strong interaction between lignin and AuNPs, enhancing the electrical conductivity of the final composite [25]. Lignin contributes not only as a structural component but also as an active layer for gas interaction. Its abundance of hydroxyl, methoxyl and aromatic groups facilitates the adsorption of polar gases, such as amines and ammonia, through hydrogen bonding, π–π interactions, and Van der Waals forces [26,27]. The resulting PLA/AuLNP composite exhibited a high affinity for volatile amines, particularly DMA, with a limit of detection (LOD) of 0.44 parts per million (ppm), demonstrating both sensitivity and selectivity in challenging environments. Notably, the sensor is able to operate at room temperature and benefits from ambient humidity, which promotes amine protonation and enhances interaction with the sensing surface, a mechanism rarely explored in lignin-based sensors [28]. These findings support the hypothesis that lignin-based nanocomposites, when rationally engineered, can serve as viable, eco-friendly alternatives to conventional sensing materials. Therefore, this work introduces a novel, green, and scalable approach to fabricate amine sensors that combine low-cost fabrication, high selectivity for dimethylamine, and complete biodegradability, a combination not previously reported in the literature.

## 2. Materials and Methods

### 2.1. Materials

The utilised solvents and polymers for the material preparations and vapour tests were as follows: Polylactic acid (PLA, Mw 230k) was purchased from GoodFellow (Cambridge, UK). Alkali lignin (Kraft Lignin), Acetone (99.8%), 2,2,2-Trifluoroethanol (TFE) ≥99%(GC), Isopropyl alcohol (99.5%), Methylamine solution 40 wt. % in H_2_O (Mw 31.06), Isobutylamine 99% (Mw 73.14), Ethylamine 70% aqueous solution, Propylamine 98% (Mw 59.11), Acetic Acid ≥ 99.7%, Ammonium hydroxide solution 28.0–30.0%, Hydrogen peroxide solution 30 wt.% in H_2_O, and tetrachloroauric acid trihydrate (HauCl_4_∙3H_2_O) (Mw 339.79) were purchased from MercK KGaA (Damstadt, Germany). All solvents were of analytical grade and used as received, without further purification.

These compounds were handled under controlled conditions in accordance with safety protocols.

Interdigitated electrodes (IDEs) supplied by Micrux Technologies (Gijón, Spain) were fabricated on borosilicate glass substrates (10 mm × 6 mm × 0.75 mm). The Pt/Ti electrodes consisted of 120 pairs, each 10 μm wide, 3500 μm long, and 150 nm thick, with a 10 μm inter-electrode gap. Prior to use, the IDE surfaces were cleaned by rinsing with soap and water, followed by immersion in a “base piranha” solution (a 3:1 *v*/*v* mixture of ammonia and hydrogen peroxide) at 60 °C for approximately 60 min under a well-ventilated fume hood. The electrodes were then thoroughly rinsed with Milli-Q water (~18 MΩ·cm), washed with isopropyl alcohol and dried under a nitrogen stream.

Lignin nanoparticles were prepared using a bottom-up approach previously reported by the authors [22] and finally incorporated into PLA nanofibres by electrospinning technology following Au^0^ decoration treatment.

Indeed, an aliquot of HAuCl_4_ was added to the aqueous suspension of lignin nanoparticles (7.1 mg/mL) to achieve a mass ratio of 0.0012:1 (*w*:*w*), and the mixture was stirred for 4 h (T: 40 °C). Taking advantage of the intrinsic reducing properties of lignin, the gold salt was reduced to metallic gold, as indicated by the reaction solution turning pink. The resulting aqueous suspension was then centrifuged three times at 9000 rpm for 20 min at 8 °C in water (NEYA 16R, REMI Elektrotechnik LTD, Vasai-401208, Palghar, Maharashtra, India) to remove any unreacted gold salt or Au^0^ in solution that were not adsorbed onto the LNPs.

### 2.2. Sensing Materials Preparations

The nanofibrous mat was fabricated using the electrospinning technology, a versatile and efficient technique capable of producing fibres with nanometric diameters [29,30,31]. This method relies on the application of a high-voltage electric field to a polymer solution, which induces the formation of continuous fibres through the controlled extrusion of a charged jet from a syringe needle. In this study, nanofibres were produced using a Fluidnatek^®^ LE-50 electrospinning platform (Bioinicia, Paterna, Valencia, Spain).

Electrospinning was performed directly onto IDEs. PLA was chosen as the polymeric matrix to incorporate the LNPs. PLA is particularly attractive for developing nanofibrous sensors due to a combination of material properties, processability, and environmental advantages [32,33]. It readily dissolves in common solvents and forms stable jets under electrospinning conditions, enabling the fabrication of uniform nanofibres with tunable diameters and porosities (key features for sensors with high surface-to-volume ratios). Furthermore, PLA exhibits good mechanical integrity, which helps maintain the structural stability of sensor platforms during handling and deployment while also withstanding moderate thermal fluctuations. As a biodegradable polyester derived from renewable resources (e.g., corn starch, sugarcane), PLA is ideal for environmentally friendly or disposable sensors.

A polymeric solution was prepared by dissolving 170 mg/mL of PLA in TFE under continuous stirring at 40 °C for a minimum of 6 h to ensure complete homogenization.

Electrospinning deposition was performed with a voltage of +7 kV applied to the needle, while the collector was maintained at −2 kV. The polymer solution was dispensed at a flow rate of 350 µL/h, with a tip-to-collector distance of 15 cm. Electrospinning was conducted under controlled environmental conditions (20 °C, 40% relative humidity), using a rotating drum collector set at 510 rpm, onto which the IDEs were fixed. Under these conditions, a uniform and reproducible deposition of nanofibres was achieved. A deposition time of 5 min was sufficient to form a homogeneous fibrous layer across the surface of the IDEs.

After fibre deposition, a dip-coating treatment with the AuLNPs solution (described in the Section 2) was applied. This step enriched the fibre surface with additional lignin nanoparticles, enhancing analyte interaction and overall sensitivity. Furthermore, the gold nanoparticles decoration was expected to improve the electrical conductivity of the sensing layer and contribute catalytic activity during the sensing process.

### 2.3. Sensing Material Characterization

**Figure 1:**
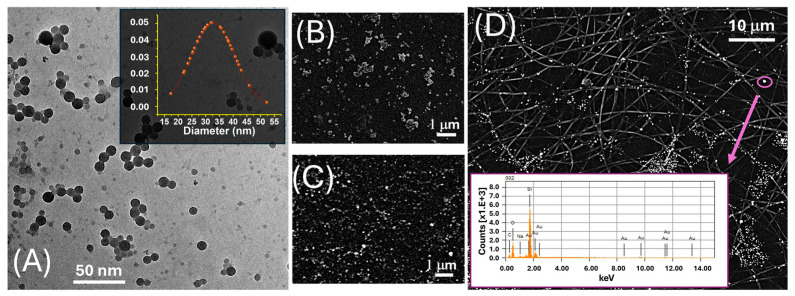
(**A**) TEM image of LNPs. The inset displays the corresponding Gaussian distribution of the particle diameter, centred around ~30 nm; (**B**) SEM image acquired in SE mode, highlighting the surface morphology of drop-cast AuLNPs; (**C**) SEM image acquired in BSE mode on the same sample, enhancing contrast for the high atomic number gold-containing nanoparticles; (**D**) SEM-BSE image of electrospun PLA fibres functionalized with AuLNPs. The bright spots represent AuLNP distribution across the fibre mat. The inset shows the EDS spectrum collected from the highlighted spot (purple circle): Mα at ~2.12 keV, Lα at ~9.71 keV, Lβ at ~11.44 keV, and Lγ at ~13.38 keV.

The morphological and compositional features of LNPs were assessed by transmission electron microscopy (TEM) (Figure 1A). Images were acquired using a CRYO-TEM TOMO TECNAI G2-20 microscope (FEI Company, Hillsboro, OR 97124-5793 USA), equipped with a LaB_6_ thermionic emission gun and a 2HS Eagle CCD camera, operating at 200 kV. Samples were prepared by depositing 3 μL of the nanoparticle suspension onto a carbon-coated copper grid, followed by air drying prior to analysis. The morphology of AuLNPs was investigated by scanning electron microscopy (SEM, JEOL JSM-6010LA; JEOL Ltd., Tokyo, Japan), as shown in Figure 1, using both secondary electron (SE, 5 kV, Figure 1B) and backscattered electron (BSE, 20 kV, Figure 1C) imaging modes. Samples were mounted on aluminium stubs using conductive carbon tape and sputter-coated with a 2 nm gold layer (Quorum Q150R ES, Quorum Technologies, East Sussex, UK) to enhance surface conductivity. Elemental analysis was performed by energy-dispersive X-ray spectroscopy (EDS) using the integrated detector, operated at 20 kV with a 60 s acquisition time (inset Figure 1D).

The TEM image in Figure 1A revealed that the LNPs possessed a predominantly spherical morphology with a relatively narrow size distribution. The LNPs appeared well dispersed, although occasional agglomeration was evident, likely attributable to capillary forces during the drying stage of the sample preparation. The inset in the upper right corner displays the particle size distribution histogram, obtained from statistical analysis of over 50 individual nanoparticles. The data fit a near-Gaussian profile, yielding an average diameter of 33 ± 8 nm. The observed size uniformity indicates a high degree of reproducibility and synthetic control in the nanoprecipitation protocol employed for LNP fabrication.

To investigate the elemental composition and spatial distribution of AuLNPs both prior to and following their integration into polymeric substrates, SEM was performed in SE and BSE modes, combined with EDS. While the resolution of SEM was insufficient to resolve individual gold nanostructures (Figure 1B), SE images revealed persistent, rounded, particle-like features, suggesting that the native morphology of the lignin nanoparticles was largely retained after metallization. BSE imaging (Figure 1C) showed discrete regions of enhanced contrast, typical of elements with high atomic numbers, such as gold, indicating the successful formation of metallic Au^0^ domains within the hybrid nanostructures.

**Figure 2:**
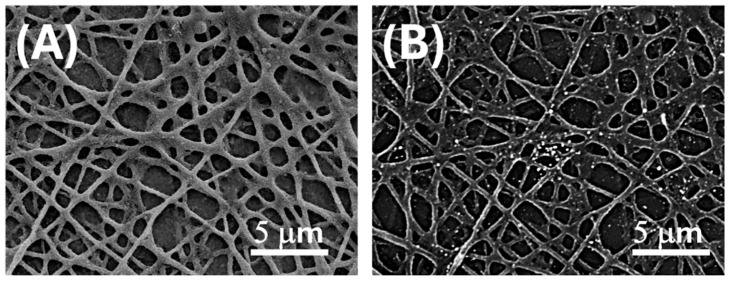
SEM micrographs of electrospun PLA fibres after surface functionalization with AuLNPs via dipping deposition. (**A**) SE image enhancing the distribution of AuLNPs across the fibre surfaces; (**B**) BSE image highlighting the presence of gold through bright contrast spots associated with higher atomic number elements.

This interpretation was supported by EDS spectra acquired from these bright regions (Figure 1D, inset), which confirmed the presence of gold, alongside carbon and oxygen signals associated with the organic PLA matrix. Upon deposition onto electrospun PLA fibres, as shown in Figure 1D and Figure 2B, the AuLNPs appeared as bright spots distributed along the fibre surfaces. EDS analysis of selected fibre regions further confirmed the presence of metallic gold through its characteristic peaks. Additional contributions from carbon and oxygen were attributed to both PLA and lignin, while silicon signals originated from the underlying borosilicate substrate used during sample preparation.

To quantify the spatial distribution of AuLNPs on the PLA nanofibres, image analysis was carried out on representative BSE-SEM micrographs using the open-source software ImageJ (version 1.53t, National Institutes of Health, Bethesda, MD, USA) in combination with the Diameter J 1.0.18 plugin. Grayscale images were converted to an 8-bit format and subjected to a brightness thresholding procedure to isolate high-intensity regions corresponding to gold-enriched domains. The estimated coverage of bright Au-containing regions on the nanofibre surface was approximately 4%.

**Figure 3:**
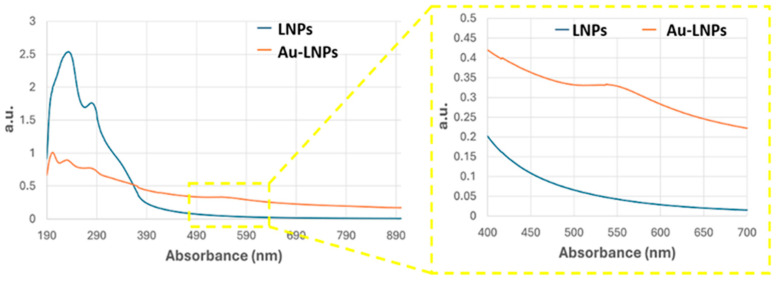
UV–Vis absorbance spectra of LNPs (blue curve) and AuLNPs (orange curve). The inset highlights the visible region (400–700 nm), where the broad absorbance feature of AuLNPs indicates the presence of nanostructured gold through surface plasmon resonance (SPR) effects.

The electrospun PLA mats (Figure 2A) exhibited a moderately porous architecture, with a mean pore area of 0.56 ± 0.66 µm^2^, indicative of a heterogeneous distribution of pore sizes. These findings are consistent with a partially and selectively decorated nanofibrous scaffold, supporting both the morphological evidence and the broad localized surface plasmon resonance (LSPR) features observed in subsequent UV–Vis spectroscopic analyses (Figure 3).

UV–Vis spectra of the AuLNP suspension (Figure 3, orange curve), compared with that of native LNPs (blue curve), revealed significant spectral changes. While the native LNPs showed intense absorption in the UV range (200–300 nm), characterized by a peak around 220–230 nm and a shoulder near 280 nm—attributed, respectively, to π→π* transitions of aromatic rings and n→π* transitions of carbonyl groups [34,35] —the AuLNPs exhibited a distinct reduction in UV absorbance intensity and the emergence of a broad absorption band extending into the visible range (500–600 nm). This feature, more evident in the magnified inset of Figure 3, is indicative of LSPR phenomena arising from metallic gold domains embedded within the hybrid particles. The absence of a sharp LSPR peak around 520 nm—commonly observed for well-dispersed, spherical AuNPs—suggests a heterogeneous size distribution and possibly non-spherical or partially aggregated gold domains, consistent with gold nucleation on biopolymer scaffolds [36,37].

The position and shape of the LSPR band imply the formation of gold nanostructures in the 20–50 nm range, in line with SEM-BSE and EDS results. Although direct visualization by TEM was not feasible for these samples, the combined evidence from SEM contrast, elemental mapping, and UV–Vis spectroscopy strongly supports a model in which gold nucleates and grows on the LNP surface during HauCl_4_ reduction. This process is likely mediated by lignin’s phenolic and carboxylic groups, which act as both reducing and stabilizing agents.

Overall, these results confirm the successful fabrication of Au-decorated lignin nanoparticles and their effective integration into PLA fibre substrates, with evidence of both surface-bound dispersion and localized aggregation, depending on deposition conditions.

Although only ~4% of the nanofibre surface appears covered by bright features in BSE-SEM, the underlying LNPs coating is substantially more extensive (Figure 2A). This suggests that most fibres are decorated with AuLNPs, even though gold nucleation or reduction occurred only partially or heterogeneously across the LNPs. As a result, the fibres retain a high degree of morphological coverage by the hybrid nanostructures, while plasmonically active gold domains remain sparsely but selectively distributed. This is consistent with the UV–Vis spectroscopy of AuLNPs performed in solution, which revealed a broad absorption band in the visible region (500–600 nm).

**Figure 4:**
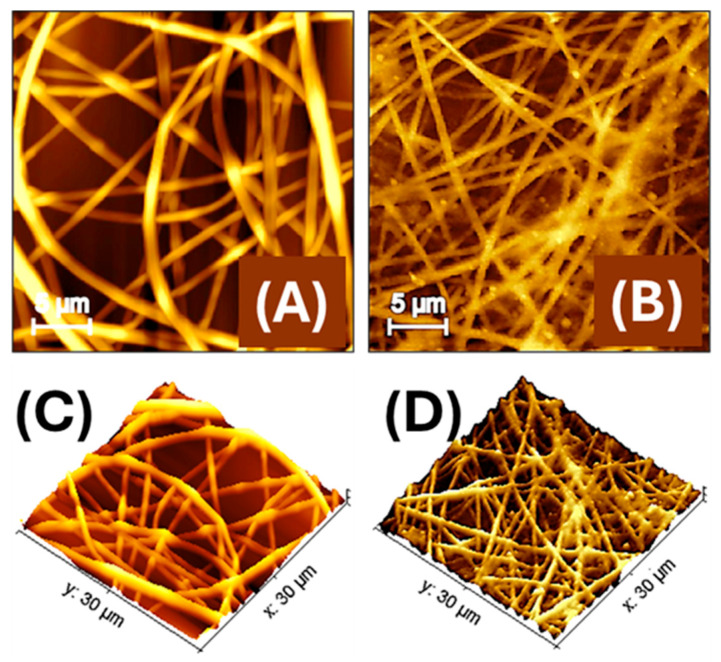
Atomic force microscopy (AFM) images of electrospun polylactic acid nanofibres (PLA-NFs) housing AuLNPs (**A**) and then decorated by dipping with AuLNPs (**B**). A 3D-topographical representation of both images is shown below (**C**,**D**).

In order to estimate the surface area available for functional interactions, we complemented SEM morphological analysis with atomic force microscopy (AFM) topographic measurements on representative areas of electrospun PLA fibres functionalised with AuLNPs, (Figure 4).

AFM images compare the morphology of electrospun PLA nanofibres embedding LNPs (Figure 4A) and those subsequently decorated via dip coating with AuLNPs, (Figure 4B). In Figure 4A the PLA nanofibres exhibit a generally continuous structure, yet with surface irregularities along the fibre axis, such as alternating swellings and constrictions, with diameters ranging approximately from 250 to 700 nm. These morphological variations are likely due to the presence of LNPs embedded within the polymer matrix, which may interfere with uniform jet stretching during electrospinning. In Figure 4B, the dip-coating process with AuLNPs induces a noticeable reorganization of the fibre morphology. Following the dip-coating treatment with AuLNPs, the fibres appear more linear and aligned, with reduced surface irregularities and a more homogeneous morphology. The apparent fibre diameter is slightly narrower, ranging from about 250 to 500 nm. This effect is not necessarily due to a radial shrinkage but results from a mild axial relaxation of the fibre structure induced by solvent interaction during the dip-coating process. This relaxation may lead to a local redistribution of polymer chains and a reduction in internal stress, resulting in straighter fibres with fewer bulges. Additionally, the fibre surfaces look densely decorated with nanoparticles of variable size, consistent with the heterogeneous deposition of AuLNP aggregates, which increases the nanoscale roughness and enhances the fibre’s functional surface area for sensing applications [38].

The data were processed using Gwyddion software (version 2.65, 2024), an open-source platform for scanning probe microscopy (SPM) data analysis and visualization [39]. AFM measurements (Figure 4B) on a representative 100 µm^2^ region yielded a real surface area of 124.6 µm^2^, corresponding to a surface increase of approximately 24.6% over the 2D projected area. This enhancement is consistent with the nanometric surface features induced by the presence of AuLNPs. The average roughness (Sa) and root-mean-square roughness (Sq) were 123.2 nm and 153.5 nm, respectively. The difference between these values reflects a surface morphology characterized by protrusions and depressions, which contribute to the increased topographical complexity. While the roughness is significant on the nanoscale, it remains compatible with the observed ~25% increase in actual surface area on the micrometric scale. The estimated fibre surface coverage of about 80%, calculated from AFM data, confirmed the formation of a moderately dense and interconnected nanofibre network across the substrate.

Figure 4C,D show 3D AFM reconstructions of the nanofibre mats before (C) and after (D) dip coating with AuLNPs. More specifically, in Figure 4C, the PLA fibres appear randomly oriented and significantly interwoven, with evident variations in height and some surface irregularities along the fibre axes. The topography reveals a complex, entangled structure, where fibre intersections create uneven elevation profiles. The surface roughness is moderate and primarily intrinsic to the polymer and embedded LNPs.

In contrast, Figure 4D reveals a more organized and coherent fibre network following the dip-coating process. Although fibre overlap is still present, the overall topography appears smoother and more continuous, with reduced fluctuations in height across the mat. The fibre surfaces exhibit fine-scale roughness due to the presence of AuLNP clusters, but the general alignment and packing of fibres seem improved.

### 2.4. Measurement System Setup

**Figure 5:**
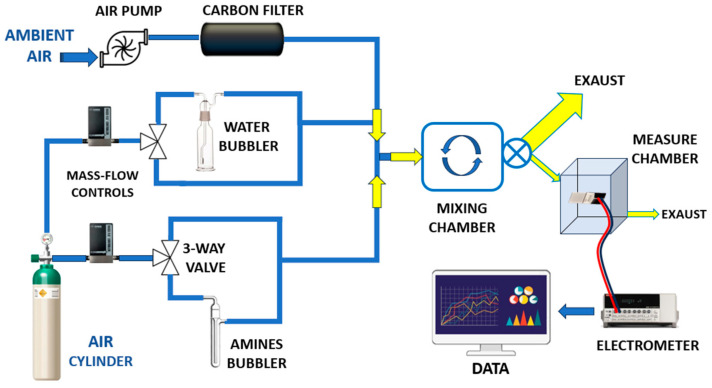
Overall view of the customized vapour generation and measurement system.

All measurements were carried out at room temperature (20 ± 2 °C) using a custom-designed gas exposure and measurement system (Figure 5).

The setup was specifically designed to ensure stable and reproducible exposure conditions while enabling the precise generation of low-ppm concentrations of the target vapour. A carrier flow of ambient air at 4000 sccms (standard cubic centimetres per minute), supplied by an air pump, was employed. Prior to entering the system, the air stream was filtered through a dust filter followed by an activated carbon filter to remove particulates, hydrocarbons, and residual VOCs.

This high flow rate was essential to dilute a small volume (typically a few sccms) of concentrated amine vapour, allowing accurate control of the final vapour concentration. Humidity regulation was achieved using a mass flow controller (MFC), which enabled the precise mixing of dry and humidified air, thereby maintaining a constant and adjustable relative humidity (RH) level.

In a parallel branch of the system, a dry carrier gas (air) from a pressurized cylinder was regulated by a separate mass flow controller (MFC) and directed toward a three-way solenoid valve. This configuration enabled either the exclusion of vapour or the metered introduction of concentrated amine vapour into the main airflow. By injecting only small fractions of vapour, the system produced reproducible and well-diluted mixtures at target concentrations. All flow streams were merged using Y-connectors and introduced into a mixing chamber (approximately 2 L in volume) to ensure homogeneous vapour distribution. A continuous sampling flow of 100 sccms was drawn from the mixing chamber by a secondary pump and directed into the sensing chamber for analysis. A key advantage of this system was the use of ambient air as the carrier gas, allowing sensor testing under conditions that more closely resemble real-world human exposure. Unlike conventional systems that often employ dry air to minimize humidity interference, this setup allowed controlled adjustment of relative humidity (RH), enabling the simulation of realistic environmental conditions. Electrical measurements of the IDE sensors in response to varying vapour concentrations were carried out using a Keithley 6517 electrometer (Solon, OH, USA). The output signals were recorded and processed using custom LabVIEW software (LabVIEW 2014, National Instruments, Austin, TX, USA), allowing real-time visualization and analysis of the sensor responses.

### 2.5. Electrical Parameters Characterization

To assess the electrical properties of the developed fibrous materials, preliminary current–voltage (I–V) measurements were conducted to characterize their conductivity and establish a baseline for comparison.

**Figure 6:**
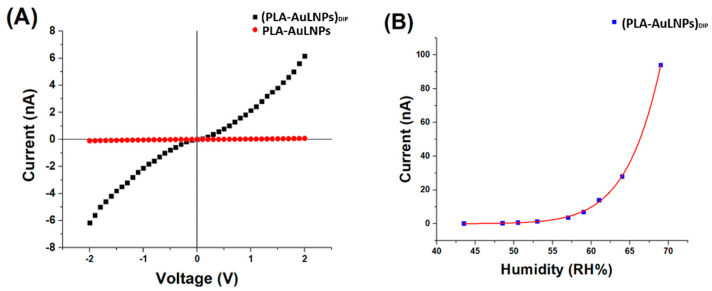
(**A**) IV curves of PLA-AuLNPs (red) and dipped fibres (PLA–AuLNPs)_DIP_ (black); (**B**) (PLA-AuLNP)_DIP_ current changes to increasing humidity.

Two sets of samples were analysed. The first consisted of electrospun PLA nanofibres embedded with gold–lignin nanoparticles (PLA–AuLNPs), tested in their as-prepared state to evaluate intrinsic electrical properties. The second set comprised the same PLA–AuLNP mats after surface functionalization via dip coating in a solution containing AuLNPs, intended to enhance surface conductivity; these samples are referred to as (PLA–AuLNPs)_DIP_, (Figure 6A).

As shown in Figure 6A, the I–V curves of the two sample types exhibit markedly different behaviours. The red curve, corresponding to the as-prepared PLA nanofibres containing embedded nanoparticles, shows a negligible current across the −2 V to +2 V range, confirming persistent insulating behaviour and the absence of significant intrinsic conductivity. In contrast, the black curve, representing the fibres after dip coating with AuLNPs, displays a symmetric yet nonlinear current response, suggestive of semiconducting or hybrid conductive behaviour.

At an applied voltage of 2 V, the PLA-AuLNP fibres exhibit a current of approximately 0.1 nA, corresponding to a resistance of ~20 GΩ.

After dip coating, the current increases to ~6.2 nA, yielding a significantly lower resistance of ~322 MΩ. According to Ohm’s law, Equation (1):

(1) $$R=\frac{V}{I}$$

and the definition of conductance, Equation (2):

(2) $$G=\frac{1}{R}$$

are used to calculate the conductance of the dipped fibres at 2 V as ~3.1 nS.

This substantial increase in current flow should confirm the effective adhesion of conductive pathways, attributed to the presence of gold nanoparticles on the fibre surface and more contact with the microelectrodes.

The nearly symmetric, nonlinear shape of the I–V curve for the dip-coated fibres suggests a non-ohmic conduction mechanism, likely governed by charge transport via hopping or tunnelling through localized states within the hybrid material. These findings indicate that the dip-coating process successfully imparts new electrical functionality to the PLA nanofibres, making the composite material a promising candidate for sensing or flexible electronic applications [40,41].

Instead, the symmetry of the IV curves around the origin indicates an absence of clear rectifying (diode-like) behaviour. This suggests that at this level of current, the contacts between fibres and fibre–metal electrodes do not form a Schottky barrier, leading to a symmetric response [42,43].

To investigate the sensor’s electrical response as a function of relative humidity (RH), the electrical current at a fixed voltage was recorded across different RH levels, ranging from 43.5% to 69% at 20 °C. The results are presented in Figure 6B. The current response increased steadily with RH, displaying an approximately linear trend up to ~60% RH. Beyond this threshold, the current exhibited a pronounced exponential increase.

This two-regime behaviour can be interpreted by considering the distinct roles of the composite components:

(i) PLA acts as the structural matrix of the nanofibres. As a hydrophobic and electrically insulating polymer [44,45], PLA provides mechanical support but does not directly participate in charge transport. Nevertheless, its fibrous morphology and porous structure facilitate the adsorption and diffusion of water vapour onto and within the fibre network, enabling interaction with the embedded nanostructures.
(ii) LNPs exhibit intrinsic dielectric and semiconductive properties and are known to participate in charge trapping and polarization mechanisms upon exposure to moisture [46]. As RH increases, water molecules adsorb onto the lignin surface, altering its dielectric environment. This adsorption can enhance proton hopping (Grotthuss mechanism) and modulate the mobility of charge carriers, leading to increased current. Above ~60% RH, multilayer water adsorption likely occurs, creating quasi-continuous water films that enable a dramatic increase in ionic conductivity, explaining the exponential rise in current;
(iii) Au^0^ nanoclusters provide nanoscale conductive sites that lower the overall resistance of the composite. They likely form percolative conductive paths along or between fibres, enabling efficient electron transfer. Additionally, Au^0^ nanoclusters can catalyse the dissociation or polarisation of water molecules adsorbed at the fibre surface, thereby enhancing sensitivity to humidity.

Therefore, in combination, these effects lead to a humidity-dependent increase in conductivity, with the (PLA-AuLNPs)_DIP_ composite remaining relatively insensitive at lower humidity levels, but showing a pronounced and sensitive response above a critical RH threshold (~60%). These findings indicate that the electrical conductivity of the sensor is positively influenced by ambient humidity, enabling reliable sensor operation at room temperature also in moist environments. However, the nonlinear transition beyond this threshold likely corresponds to the onset of water condensation or capillary condensation within the nanofibre mat, facilitating enhanced ionic transport and signal transduction.

## 3. Results and Discussion

After completing the setup and stabilization of the sensing platform, we investigated the material’s response to selected analytes. A literature survey reported that lignocellulosic-based sensors have shown promising selectivity towards ammonia and amine compounds [19]. Lignin, following appropriate curing treatments, is frequently employed as a carbon source in gas-sensing applications. Its use is often synergistically combined with metal or metal oxide nanostructures, such as graphene oxide [21] or ZnO [47] and conductive polymers (CPs), such as polyaniline [19], to enhance the material’s sensitivity, surface area (e.g., foam, hieratic nanostructures, nanofibrils distributed in papers) and electron transfer properties. These hybrid systems exploit lignin’s intrinsic aromatic structure and functional groups, which contribute to selective adsorption and improved sensing performance, particularly for ammonia and amine vapours. Therefore, firstly, we exposed our hybrid material to different ammonia vapour concentrations (228, 152, 76 and 38 ppm, respectively) to evaluate its electrical conductivity changes.

**Figure 7:**
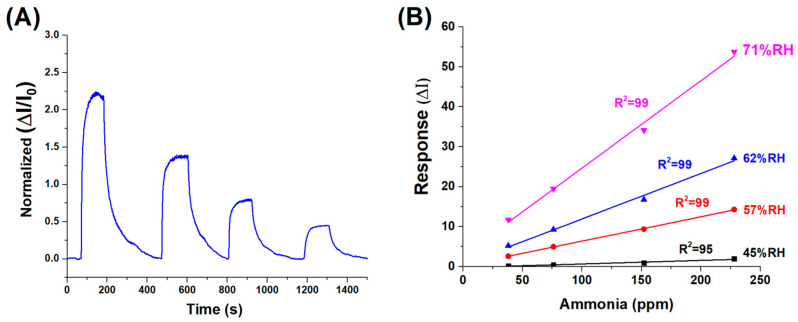
(**A**) Dynamic response of the sensor to decreasing concentrations of ammonia vapour, ranging from approximately 230 to 40 ppm (%RH:62). (**B**) Normalised sensor response curves at varying relative humidity levels (45% to 71% RH), highlighting the influence of ambient humidity on sensing performance.

From the transient sensor responses, we observed that the changes in electrical conductivity were directly and quantitatively correlated with the ammonia vapour concentrations, as shown in Figure 7A. After the signal reached a stable maximum (steady-state response), the ammonia flow was stopped and replaced with clean air. The signal gradually returned to its baseline level, indicating a reversible and repeatable sensing behaviour.

The dynamic response and recovery behaviour of the (PLA-AuLNPs)_DIP_ sensor upon exposure to varying ammonia concentrations is depicted in Figure 7A, where the normalised transient responses (ΔI/I_0_) of the current signal are reported. The response times, here measured as the time required to reach the peak signal upon exposure to the analyte (plateau), ranged from approximately 70 to 100 s across the tested concentrations. Similarly, the recovery times, calculated as the duration needed for the signal to return to its baseline after analyte removal, were comparable, ranging from 80 to 100 s. This symmetry between response and recovery times indicates a fast and reversible sensing mechanism. The consistent kinetics observed across varying concentrations suggests that the material exhibits stable adsorption–desorption dynamics with ammonia vapours, a desirable feature for real-time sensing applications.

To evaluate the influence of environmental humidity on sensor performance—an effect previously suggested by the results in Figure 6B—a series of measurements were carried out using ammonia concentrations between 230 and 40 ppm under different relative humidity (RH) conditions: 45%, 57%, 62%, and 71%. As illustrated in Figure 7B, the sensor responses at each humidity level were fitted with linear regression curves, yielding slopes of 0.0089 (R^2^ = 0.95) at 45% RH, 0.0610 (R^2^ = 0.99) at 57% RH, 0.1138 (R^2^ = 0.99) at 62% RH, and 0.2189 (R^2^ = 0.99) at 71% RH. Except for the lowest humidity condition, all data sets exhibited excellent linearity (R^2^ ≥ 0.99), confirming that both ammonia concentration and ambient humidity significantly influence the sensor’s response.

**Figure 8:**
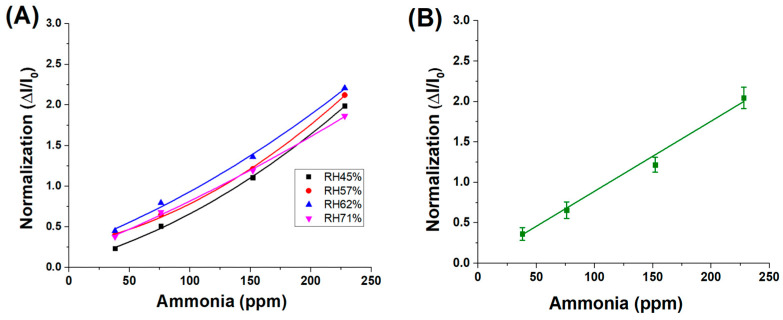
(**A**) Normalised response values of different RH% concentrations; (**B**) average fitting of the 4 normalizations.

However, when the sensor responses are normalised across all humidity levels for the same range of ammonia concentrations, the resulting data converge onto a single linear trend, as shown in Figure 8A, with an average fitting slope of approximately 9.0 × 10^−3^ ± 4.5 × 10^−4^, as shown in Figure 8B. This indicates that, although absolute current responses are influenced by ambient humidity—likely due to variations in surface conductivity or analyte adsorption—the normalised signal (ΔI/I_0_) effectively compensates for these effects. As a result, the sensor’s sensitivity to ammonia remains consistent across different environmental conditions, suggesting the robustness and reliability of the normalised response metric.

These findings suggest that the normalised sensitivity of the material to ammonia vapours remains unaffected by variations in relative humidity. The observed increase in the absolute sensor response under higher humidity conditions is therefore attributed to an amplifying effect induced by water molecules, likely related to enhanced ionic conductivity or facilitated analyte adsorption at the sensor surface.

**Figure 9:**
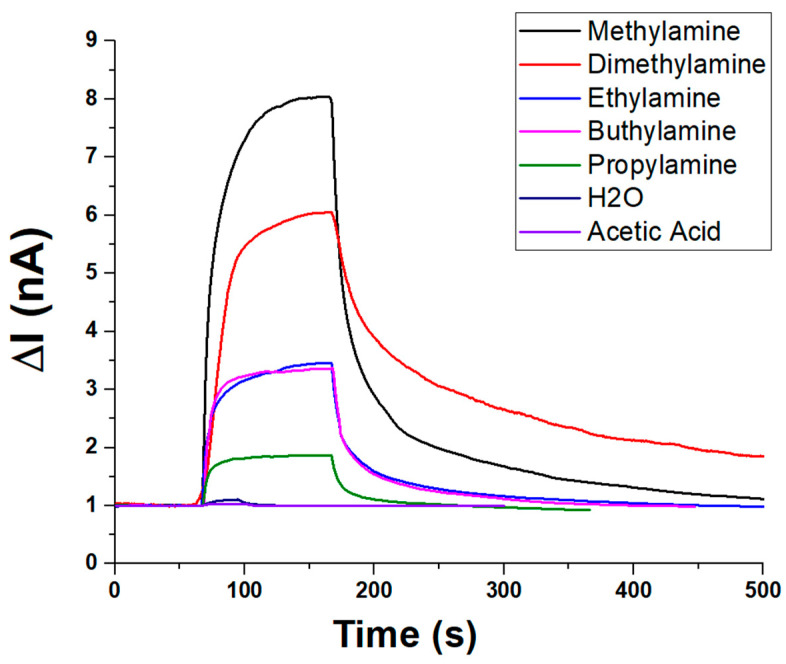
Plot overlapping the transient (PLA-AuLNPs)_DIP_ sensor responses to different vapour chemicals.

In addition to ammonia, the sensor’s selectivity toward amines and VOCs was evaluated under constant humidity (about 55% RH) and temperature (20 °C) conditions. The sensor’s dynamic response toward various amines and VOCs is depicted in Figure 9. Upon exposure, all analytes induced a rapid increase in current (ΔI), followed by a plateau and a slower recovery phase, indicating fast adsorption kinetics and comparatively slower desorption. Among the tested compounds, methylamine elicited the highest response (~8.5 nA, V:1.5 V), followed by dimethylamine (~6.5 nA), suggesting strong interactions with the sensing material. Moderate responses were recorded for n-buthylamine and ethylamine (ranging from ~3 to 3.5 nA), while propylamine yielded a lower signal (~2 nA), likely due to steric or diffusional effects. In contrast, water vapour and acetic acid produced minimal current variations (~1 nA), underscoring the sensor’s selectivity toward amines and its low susceptibility to humidity or acidic VOC interference. These results highlight the sensor’s potential for selective detection of low-molecular-weight amines.

To quantify the concentration of volatile organic compounds (VOCs) introduced into the sensing system, theoretical estimates were first obtained based on the saturated vapour pressure of each analyte at the working temperature (20 ± 2 °C), using standard reference data. These values were subsequently corrected by considering the relative flow rates of the saturated vapour and the carrier gas, enabling the calculation of the dilution factor. This approach allowed a reliable estimation of the final VOC concentrations reaching the sensor chamber. It is important to note that, in Figure 9, each measurement was conducted using the same volumetric aliquot of VOC delivered under a constant carrier gas flow. However, due to differences in the saturated vapour pressure of each compound, the actual concentration of VOCs in the gas phase varied accordingly. As a result, the observed sensor responses reflect not only the material’s selectivity but also the intrinsic volatility of each analyte.

Under these conditions, the sensor responses followed the decreasing trend of vapour pressure of each compound:

Methylamine > Diethylamine > Ethylamine > Propylamine > n-Butylamine

This selectivity apparently favours short-chain primary amines, but it is presumably influenced by the different vapour pressures and, thus, different actual concentrations. Negligible responses were reported to the same volume percentages of acetic acid and water vapour.

**Figure 10:**
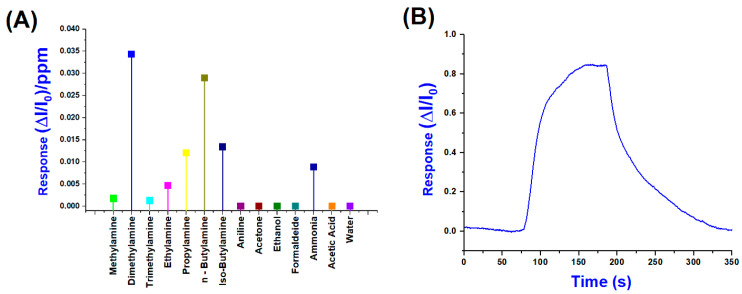
(**A**) Normalised response values per ppm of (PLA-AuLNPs)_DIP_ sensor; (**B**) a normalised transient sensor response to DMA (25 ppm) (**B**).

Conversely, Figure 10A shows the normalised sensor responses (ΔI/I_0_)/ppm to a range of VOCs, including primary, secondary and tertiary amines, as well as potential interferents, such as water, ethanol, acetone, formaldehyde, and acetic acid. Among all the tested analytes, dimethylamine and n-butylamine produced the highest responses, exceeding 3 × 10^−2^ (ΔI/I_0_)/ppm, suggesting a high affinity and strong interaction with the sensing layer. According to these results, in evaluating their selectivity index, we can easily calculate a 32.6% response for DMA and a total of 60.1% for the combined response of DMA and n-Butylamine, indicating a preferential sensitivity toward these amines.

Significant responses were also observed for iso-butylamine and propylamine, followed by ammonia, while lower signals were recorded for bulkier amines, such as trimethylamine and the aromatic compound aniline, likely due to steric hindrance or reduced volatility. In contrast, non-amine VOCs generated negligible responses below 5 × 10^−3^ (ΔI/I_0_)/ppm, underscoring the sensor’s selectivity toward low-molecular-weight aliphatic amines. The moderate response to ammonia, compared to organic amines, can be attributed to its inorganic nature, smaller molecular size, and distinct adsorption mechanism, which may involve weaker interactions with the sensing material, such as hydrogen bonding or purely physisorption processes, rather than stronger chemisorption observed for aliphatic amines. In contrast, organic amines contain alkyl groups that enhance van der Waals forces and hydrophobic interactions, facilitating more robust adsorption onto the sensor surface. This difference in interaction strength and mechanism is well documented in the gas-sensing literature, particularly for metal oxide and polymer-based sensors, where ammonia often shows distinct binding profiles compared to alkylamines. For instance, Rodríguez et al. (2020) demonstrated that covalent organic framework (COF) films exhibit higher sensitivity and selectivity toward low-molecular-weight aliphatic amines than ammonia, highlighting the role of molecular structure in sensor–analyte interactions. These findings confirm the sensor’s strong potential for selective detection of amine vapours and demonstrate its suitability for monitoring complex gas mixtures [48].

Figure 10B shows the dynamic and normalised response of the sensor upon exposure to 25 ppm of DMA, which elicited the highest signal among the tested analytes. The response exhibits a sharp rise beginning (around 80 s), reaching a maximum normalised signal (ΔI/I_0_) close to 0.85 within approximately 120 s of exposure. Upon cessation of the DMA flow (about 200 s), the signal gradually declines, demonstrating a partial recovery over the next 150 s, indicative of relatively strong and possibly semi-reversible adsorption.

The pronounced response observed for DMA can be rationalized by considering both the physicochemical properties of the analyte and the structural composition of the sensing material. DMA is a small, highly volatile secondary amine characterized by significant basicity and nucleophilicity, which promote strong interactions with the sensing surface.

In our system, the enhanced response likely originates from a synergistic contribution of the three main components: the gold-functionalized lignin nanoparticles, the PLA fibrous scaffold, and the analyte itself. Gold nanoparticles (AuNPs), deposited on the surface of lignin particles, are known to act as electron-rich sites facilitating charge transfer and adsorption processes. In particular, their soft Lewis acid character may enable soft–soft interactions with the lone pair of electrons on the DMA nitrogen, as previously reported in metal–amine sensing systems.

The lignin may play a dual role: it stabilizes the gold nanoclusters, and provides a rich chemical environment due to its hydroxyl and aromatic groups. These moieties are capable of establishing additional non-covalent interactions with amines, such as hydrogen bonding and π–π stacking. SEM observations confirmed that AuNPs are predominantly localized on the outer surface of lignin particles, thus remaining accessible to vapour-phase analytes.

As for the interaction with the PLA matrix, AuLNPs are not covalently bonded but become physically entrapped and partially embedded within the fibres during the electrospinning process. Additionally, surface decoration is achieved through a subsequent dip-coating step, resulting in external deposition of AuLNPs onto the nanofibre mat. This dual integration approach ensures both internal incorporation and surface localization of the functional nanoparticles. The physical entrapment during electrospinning provides mechanical anchoring and intimate interfacial contact, likely mediated by Van der Waals forces and potential hydrogen bonding between the ester groups of PLA and the functional groups present in lignin. The fibrous morphology of PLA is further expected to enhance sensor performance by offering a porous architecture with high surface area, which facilitates analyte diffusion, adsorption, and retention.

DMA’s relatively low steric hindrance and high vapour pressure contribute to its fast transport through the PLA mesh, increasing the likelihood of interaction with the active Au-lignin sites. These combined effects account for the selective and sensitive detection of DMA, even at low concentrations.

DMA is a key chemical intermediate in the production of various industrial compounds, including dimethylhydrazine, dimethylformamide and dimethylacetamide. It is widely used in the manufacture of pesticides, pharmaceuticals, dyes, rubber, plastics, surfactants, and rocket fuels. Given the volatile and irritant nature of DMA, rapid detection is crucial, particularly in industrial processes involving rubber, dyes, or agricultural chemicals, where accidental releases or leaks can occur. Based on these measurements, and applying the three-times-the-signal-to-noise criterion using the standard deviation of the baseline signal [49,50], a LOD of 0.44 ppm was determined for DMA. Similarly, we also calculated the limit of quantification (LOQ), based on the ten-times signal-to-noise criterion, which was found to be 1.44 ppm.

This value is significantly lower than the current Occupational Safety and Health Administration Permissible Exposure Limit (OSHA-PEL) of 10 ppm [51], highlighting the sensor’s potential for monitoring DMA in occupational settings. Although various analytical techniques have been developed to detect DMA, such as spectrophotometry, liquid and gas chromatography, and capillary electrophoresis, these methods require complex, costly instrumentation and are not suitable for in-field or real-time applications [52,53,54,55,56]. Metal oxide semiconductor (MOS)-based sensors have demonstrated good sensitivity and selectivity toward DMA, although they typically operate at high temperatures. For instance, ZnO nanostructures were shown to detect DMA at 370 °C [57]. Pd-modified ZnO films displayed higher responses to DMA than trimethylamine at 300 °C [58] and MgO-doped In_2_O_3_ loaded with Pt showed enhanced response at 300 °C [59]. More recent designs, such as the ZnO-carbon nitride (ZOCN) composite, achieved an LOD of 920 ppb at 140 °C after calcination at 550 °C [60], while ZnO nanograin-based sensors reported an impressive LOD of 108 ppb, albeit at an operating temperature of 240 °C [61]. TiO_2_ nanotube sensors doped with Nb also demonstrated linear sensitivity in the 0–50 ppm range at 300 °C [62]. Some room-temperature MOS sensors based on nanostructures have been reported [63,64], but they typically operate in higher concentration ranges due to lower sensitivity. For instance, β-Bi_2_O_3_ thin films exhibited measurable resistance changes in the 0.5–100 ppm range of DMA under dry air and room-temperature conditions [65].

Among these, the (PLA-AuLNPs)_DIP_ sensor offers several advantages: room-temperature operation, low energy consumption, user friendliness, and a compact design suitable for portable systems. Most importantly, it is entirely fabricated from biodegradable and renewable materials, namely, lignin derived from agro-industrial waste and polylactic acid (PLA), offering a sustainable alternative to conventional systems.

**Figure 11:**
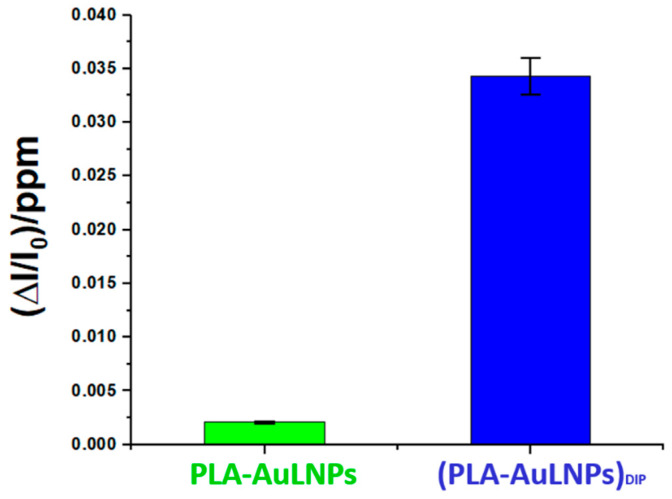
Comparison of the normalised PLA-AuNPs and (PLA-AuNPs)_DIP_ sensors’ responses to 1 ppm of DMA.

To elucidate the role of AuLNPs in selective DMA detection, sensors with and without external AuLNP functionalization were tested under controlled conditions (20 °C, 56% RH). As shown in Figure 11, the composite (PLA-AuLNPs)_DIP_ sensor displayed a response approximately 17 times higher than that of the PLA sensor encapsulating AuLNPs, confirming the benefit of surface exposure. Error bars indicate good measurement reproducibility.

The sensing mechanism benefits from the high surface-to-volume ratio of electrospun PLA nanofibres, which enhance the exposure of AuLNPs to gas molecules. AuNPs improve the electrical conductivity and introduce catalytic sites for electron transfer, while lignin contributes hydroxyl and aromatic groups capable of interacting with amines via hydrogen bonding and weak van der Waals forces. These interactions are strengthened under humid conditions due to the protonation of amines and ammonia, enhancing their affinity for the gold–lignin interface and facilitating charge transfer.

Overall, the synergy between PLA, lignin, and AuNPs provides a sensitive, selective, and environmentally friendly platform for detecting amine vapours such as DMA at room temperature.

The effect of temperature on the sensor response was not addressed in the present study, as the experimental design was specifically aimed at evaluating sensor performance under ambient conditions and varying levels of relative humidity. Nonetheless, temperature is recognized as a critical factor influencing gas adsorption/desorption dynamics and long-term sensor stability.

Previous studies have indicated that elevated temperatures can enhance the mobility of polymer chains in PLA nanofibres, potentially altering their porosity and mechanical properties, which may in turn influence analyte diffusion and interaction at the sensing interface [66].

Similarly, lignin-based materials are reported to undergo structural transitions or softening at higher temperatures, which could affect their adsorption capacity and electron transfer behaviour, particularly in hybrid systems incorporating metal/CNTs/graphene nanostructures [67].

## 4. Conclusions

This study demonstrated the development and characterization of a biodegradable sensor based on electrospun PLA nanofibres functionalized with AuLNPs for the detection of volatile amines, with enhanced selectivity toward dimethylamine (DMA) vapours. The sensor exhibited a LOD of approximately 440 ppb under ambient conditions, maintaining a stable signal-to-noise ratio. These results are particularly relevant when compared to conventional metal oxide semiconductor (MOS)-based sensors, which often require elevated operating temperatures (≥240 °C) and greater energy consumption.

Relative humidity (RH) was found to significantly influence sensor performance by facilitating amine protonation, which in turn enhanced charge transfer and interaction with the sensing surface. This effect underscores the importance of humidity control in optimizing sensitivity and selectivity under realistic ambient conditions.

Comprehensive morphological and compositional characterizations using SEM (SE and BSE modes), EDS, and AFM confirmed the successful integration of AuLNPs within and on the surface of the PLA nanofibres. Backscattered electron imaging revealed the presence of localized high-atomic-number domains, attributed to gold. Nevertheless, the coverage highlights also the need for process optimization in the metallization step, to enhance the formation and distribution of metallic gold on lignin nanoparticles. Future work will include the use of high-resolution transmission electron microscopy (HRTEM) and related techniques to elucidate the structural relationship between gold and the lignin matrix at the nanoscale. The porous fibrous structure of electrospun PLA facilitated analyte diffusion and retention, while the dual incorporation of AuLNPs, both embedded during electrospinning and applied by post-process dip coating, was essential for imparting hybrid electrical properties and enhancing sensitivity. Electrical characterizations showed that dip-coated samples exhibited semiconductive behavior, in contrast to the intrinsic insulating nature of PLA-AuLNP composites without surface treatment. Importantly, the sensor is fabricated entirely from biodegradable and waste-derived components, promoting its potential for sustainable and low-impact applications in air quality monitoring, food packaging, and wearable diagnostics. The use of PLA and lignin, both abundant and renewable materials, aligns with circular economy principles and reinforces the eco-design of the sensing platform. A deep comprehension of the influence of lignin and gold nanoparticle concentration on the electrophysical and sensing properties should provide valuable insights for sensor optimization. Although this was beyond the scope of the current proof-of-concept study, it represents a critical direction for future research. Ongoing studies will focus on optimizing Au growth and deposition protocols, improving long-term stability, and expanding the detection spectrum to include a broader range of volatile amines. In parallel, efforts will be directed toward evaluating the effects of temperature and humidity on sensor performance and integrating the platform into portable, low-power devices suitable for real-world deployment.

## Data Availability

The data presented in this study are available on request from the corresponding author. The data are currently stored in IRIS, the institutional open-access repository of the National Research Council of Italy (CNR).
